# Online T group experiences during COVID-19 pandemic

**DOI:** 10.1192/j.eurpsy.2024.1038

**Published:** 2024-08-27

**Authors:** G. Arbanas, A. Botica, H. Goršić, M. Baković, M. Bagarić, I. Barun

**Affiliations:** ^1^department of forensics, University Psychiatric Hospital Vrapče, Zagreb; ^2^Department of psychiatry, University Hospital of Split, Split; ^3^University Psychiatric Hospital “Sveti Ivan”, Zagreb; ^4^Ugljan, Psychiatryc Hospital, Zadar; ^5^University Psychiatric Hospital Vrapče, Zagreb, Croatia

## Abstract

**Introduction:**

Online group therapy has become more popular in the past few years. But as a result of the COVID-19-caused pandemic, it developed suddenly. Due to the conventional face-to-face format no longer being possible and the need for psychotherapists to conduct psychotherapy online, the pandemic has had significant effects on group psychotherapy and the interactions between group therapy members. While therapists are becoming accustomed to the modern form of psychotherapy, its efficacy is being questioned due to technical issues, the problem of the therapeutic alliance, the environment, the ability to read nonverbal signals, breaking group norms, etc. Since the pandemic did not abate, as a part of specialist education training groups were also held online.

**Objectives:**

The pandemic changed the basic settings of our Group-Analytic Training Group, forcing us to switch to online sessions. This study aimed to find personal experiences that varied throughout online and face-to-face meetings.

**Methods:**

Seven out of the twelve participants accepted to take part in the group therapy/training after it was recommended by the group leader that they write a paper. After 30 sessions, the group turned from face-to-face to online group therapy, and the members were asked how they felt about the difference between the two types of therapy. A questionnaire was produced by the group’s leader and a number of other participants, who then forwarded it through email to every group member.

**Results:**

Everyone who participated thought that because one can more quickly pick up on non-verbal signs in a face-to-face scenario, it was simpler to notice feedback from the other group members. Most participant comments focused on the leader’s role. The majority of members claimed that taking part in the experiential group had benefited both their personal and professional lives.However they thought the in-person setting was better since it was more interesting and complex.

**Conclusions:**

Since there were no other options during the epidemic, group therapy has moved to virtual environments, although there are still a lot of problems to this method. The formation of group cohesion becomes difficult by the absence of group members’ physical presence and by the inability to completely understand nonverbal communication.

**Disclosure of Interest:**

None Declared

